# Esophageal microbiome signature in patients with Barrett’s esophagus and esophageal adenocarcinoma

**DOI:** 10.1371/journal.pone.0231789

**Published:** 2020-05-05

**Authors:** Loris Riccardo Lopetuso, Marco Severgnini, Silvia Pecere, Francesca Romana Ponziani, Ivo Boskoski, Alberto Larghi, Gianluca Quaranta, Luca Masucci, Gianluca Ianiro, Tania Camboni, Antonio Gasbarrini, Guido Costamagna, Clarissa Consolandi, Giovanni Cammarota

**Affiliations:** 1 Dipartimento di Scienze Mediche e Chirurgiche, UOC Medicina Interna e Gastroenterologia, Fondazione Policlinico Universitario A. Gemelli IRCCS, Roma, Italia; 2 Department of Medicine and Ageing Sciences,”G. d'Annunzio” University of Chieti-Pescara, Chieti, Italia; 3 Center for Advanced Studies and Technology (CAST), “G. d'Annunzio” University of Chieti-Pescara, Chieti, Italia; 4 Institute of Biomedical Technologies, Italian National Research Council, Segrate (Milano), Italia; 5 Digestive Endoscopy Unit, Fondazione Policlinico Universitario A. Gemelli IRCSS, Roma, Italia; 6 Centre for Endoscopic Research Therapeutic and Training CERTT, Università Cattolica del Sacro Cuore, Roma, Italia; 7 Istituto di Patologia Speciale Medica, Università Cattolica del Sacro Cuore, Roma, Italia; 8 Dipartimento di Microbiologia, Fondazione Policlinico Universitario A. Gemelli IRCCS, Roma, Italia; Peter MacCallum Cancer Centre, AUSTRALIA

## Abstract

Preliminary studies suggested a possible correlation of microbiota with Barrett’s esophagus (BE) and esophageal adenocarcinoma (EAC), where the need for tools to ameliorate its poor prognosis is mandatory. We explored the potential signature of esophageal microbiota and its predicted functional profile along the continuous spectrum from BE to EAC. We analyzed through 16S-based amplicon sequencing the mucosal microbiota and the microbiota-related functional predictions in 10 BE and 6 EAC patients compared with 10 controls, exploring also potential differences between the metaplastic mucosa (BEM) and the adjacent normal areas of BE patients (BEU). BEM and EAC showed a higher level of α and β-diversity. BEM evidenced a decrease of *Streptococcus* and an increase of *Prevotella*, *Actinobacillus*, *Veillonella*, and *Leptotrichia*. EAC displayed a striking reduction of *Streptococcus*, with an increase of *Prevotella*, *Veillonella* and *Leptotrichia*. LefSe analysis identified *Leptotrichia* as the main taxa distinguishing EAC. BEM showed a decreased α-diversity compared with BEU and a reduction of *Bacteroidetes*, *Prevotella* and *Fusobacterium*. Functional predictions identified peculiar profiles for each group with a high potential for replication and repair in BEM; an upregulated energy, replication and signaling metabolisms, with the fatty-acids biosynthesis and nitrogen and D-alanine pathways down-regulated in EAC. Our pilot study identifies a unique microbial structure and function profile for BE and EAC, as well as for metaplastic and near-normal areas. It proposes a new concept for BE, which could be intended not only as the histological, but, also, as the microbial closest precursor of EAC. This requires further larger follow-up studies, but opens intriguing horizons towards innovative diagnostic and therapeutic options for EAC.

## Introduction

Esophageal cancer is the eight most commonly diagnosed cancer worldwide and represents one of the most frequent causes of cancer-related death [1]. The prevalent histological type in the West Countries is the adenocarcinoma (EAC) of the distal esophagus, harboring peculiar molecular characteristics, different from the squamous cell carcinoma that predominates in the Eastern Nations [2]. The only established precursor of EAC is Barrett's esophagus (BE), a condition in which the normal stratified epithelium is replaced with a metaplastic columnar layer, functioning as a protective shield against gastroesophageal acid reflux. However, persistent acid stress and consequent inflammation have been proposed to cause proliferation of BE cells that may lead to the progression towards EAC [3]. Despite the introduction of innovative therapies such as surgery, chemo- and radio-therapy, the prognosis in patients with EAC remains poor [4]. Therefore, a deeper knowledge of the pathogenesis that drives the transition from normal epithelium to BE and EAC is the cornerstone to obtain new therapeutic alternatives, improving diagnostic and prognostic tools to accurately refine the individual risk.

Interestingly, the gastrointestinal (GI) microbiota has been demonstrated to exert a crucial role in health, as well as in several GI [5–8] and extra-intestinal diseases [9–12], and, also, in various types of cancers [13, 14]. The GI microbiota is considered a dynamic system living in a synergistic relationship with its host, involved in the maintenance of immunological homeostasis. Emerging evidences have linked tumor initiation and progression in the GI tract with microbiota through DNA damage, activation of oncogenic signaling pathways, production of tumor-inducing metabolites and suppression of the immune response [14–19]. To date, important improvements in culture-independent molecular techniques have allowed the identification of the potential main bacterial actors involved in these processes, which could be useful markers to stratify cancer risk [20–23]. At the same time, since GI microbiota can be modulated by a rational application of therapeutic tools [24], a better knowledge of the relationship between BE, EAC and the microbiota may have clear clinical implications.

In the present study, we characterized the esophageal microbiota composition and inferred functional profile in patients with BE and EAC compared with control individuals. We also investigated whether microbial structure and function could differentiate the metaplastic mucosa from the adjacent normal esophageal areas in BE patients. Taken together, our data indicate that BE and EAC mucosal samples, as well as metaplastic and near-normal areas, can be differentiated by a peculiar gut microbiota profile, suggesting that it can represent one of the predisposing factors of BE, which could be not only the histological, but, also, the microbial closest precursor of EAC.

## Material and methods

### Study design and sample collection

From September 2016 to January 2018, consecutive patients with symptoms requiring upper GI endoscopy at the Fondazione Policlinico A. Gemelli in Rome were evaluated for the inclusion in this observational prospective study. Exclusion criteria were the following: previous endoscopic or surgical treatment on the stomach and/or the esophagus; active infection of the oral cavity; hepatitis B virus, hepatitis C virus or human immunodeficiency virus infection; unable to suspend anticoagulation therapy; unable to perform or intolerant to upper endoscopy; history of antibiotics and/or probiotics use in the 4 weeks preceding sampling; history of proton pump inhibitors or Histamine 2 Receptor Antagonist use in the 2 months before endoscopic biopsies.

BE was diagnosed by the presence of at least 1 cm of columnar-lined mucosa in the esophagus with the microscopical presence of intestinal metaplasia. EAC of the distal esophageal/esophagogastric junction was diagnosed histologically. During single endoscopy, bioptic mucosal samples were obtained for microbiological assessment with the following pattern:

- healthy controls (CTRL): 2 biopsies from the normal esophageal mucosa;
- patients with BE: 2 biopsies from the esophageal metaplastic lesion (BEM) and 2 from the normal esophageal mucosa (BEU);
- patients with EAC: 2 biopsies from the neoplastic lesion.

In order to avoid contamination, each biopsy was taken at the time of insertion with different disposable forceps. Samples were immediately stored at -80°C.

All enrolled subjects provided their written informed consent. The study protocol was conducted in accordance with the principles of the Declaration of Helsinki and approved by the “Università Cattolica del Sacro Cuore” Ethic Committee.

### DNA extraction and library preparation

Genomic DNA was isolated from one mucosal biopsy for each condition using High Pure PCR Template Preparation Kit (Roche, Basel, Switzerland) according to manufacturer’s instructions. 200 μl of Tissue Lysis Buffer and 40 μl of Proteinase K were added to 25–50 mg of sample material. This suspension was incubated for 3 hours at 55˚C until tissue was completely digested. Then, 200 μl of Binding Buffer were added and incubated for 10 minutes at 70˚C. Finally, samples were washed with 500 μl of Washing Buffer using a column (High pure filter tube) and eluted with 200 μl of Elution Buffer.

DNA concentration and quality were determined using a NanoDrop ND‐1000 spectrophotometer (NanoDrop Technologies, Wilmington, DE, USA) and a TapeStation 2200 (Agilent Technologies, Santa Clara, CA, USA). The V3‐V4 hypervariable regions of the 16S ribosomal RNA (rRNA) gene were amplified according to the 16S Metagenomic Sequencing Library Preparation protocol (Illumina, San Diego, CA, USA) and sequenced on a MiSeq platform (Illumina), in a single 2 × 300 bp paired-end run.

### Bioinformatic analysis

Raw sequencing reads were trimmed down to 250 nt, due to quality, and processed by merging overlapping pairs with PandaSeq software (v2.5, “PAired-eND Assembler for DNA sequences”) [25], discarding fragments of length outside 250–900 bases range, non-overlapping sequences and sequences having more than 25% nucleotides with a phred score ≤3. Quality-filtered reads were analyzed by QIIME suite (release 1.8.0) [26], grouped into OTUs (Operational Taxonomic Units) by UCLUST [27] at 97% similarity and taxonomically classified against the 13.8 release of the Greengenes database (ftp://greengenes.microbio.me/greengenes_release/) by RDP classifier [28] at 50% confidence. Singleton OTUs (i.e., clusters made up of only 1 read) were discarded as likely chimeric sequences. Sample biodiversity (*i*.*e*., α-diversity) was estimated according to different microbial diversity metrics (*i*.*e*., chao1, Shannon index, observed species and Faith’s phylogenetic distance), whereas inter-sample diversity (*i*.*e*., β-diversity) analysis was conducted using weighted and unweighted Unifrac [29] and Bray-Curtis distances and Principal Coordinates Analysis (PCoAs). Data separation was tested with a permutation test with pseudo F-ratios using "adonis" function from R package "vegan" (version 2.0–10) [30] with 999 random permutations. For beta distances evaluation, the distance between each BEM sample and the corresponding BEU sample from the same patient was calculated and compared to the median of distances between each BEM sample and the BEU samples from other individuals. When comparing paired samples, a Wilcoxon signed rank test was used for both relative abundance and beta-distances comparisons. Otherwise, for relative abundance analysis, a Mann–Whitney U-test was used. A p-value <0.05 was chosen as the threshold for statistical significance. LEfSe (Linear discriminant analysis Effect Size) [31] was employed to search for features most likely to explain differences between EAC, BEM and CTRL.

Species-level characterization of the four main genera present in the samples (*i*.*e*., *Streptococcus*, *Prevotella*, *Veillonella* and *Leptotrichia*) was performed by BLAST-aligning all reads belonging to these genera to a custom reference database of all available reference sequences in NIH-NCBI database (ftp://ftp.ncbi.nlm.nih.gov/genomes/refseq/bacteria/). Potential matches were filtered in order to retrieve an unequivocal classification for each read [32–34].

Co-abundance network analysis was performed as previously described [35], using Kendall’s correlation between taxa and building hierarchical clusters of co-abundant groups (CAGs) at genus level by Spearman's correlation metric and Ward linkage. Cytoscape (v 3.0) [36] was used to graphically represent CAGs, as well as relative abundance of bacterial genera and strength of correlation. For full description, see *Supplemental Information Appendix*, Materials and Methods.

Finally, the Phylogenetic Investigation of Communities by Reconstruction of Unobserved States (PiCRUST) [37] pipeline and the Kyoto Encyclopedia of Genes and Genomes pathways database (KEGG) [38] were used to perform a functional prediction based on the 16S taxonomic profiles of each sample.

Raw reads from this experiment are available in NCBI Short-read Archive (SRA) under accession number PRJNA553177 (https://www.ncbi.nlm.nih.gov/bioproject/PRJNA553177).

## Results

A total of 16 patients, 10 of whom with a new diagnosis of BE and 6 with a new diagnosis of distal esophageal/esophagogastric junction cancer, and 10 CTRL without any endoscopic sign of mucosal disease were enrolled. Demographic characteristics of the study population are shown in Table 1. No statistical significant age differences were found among groups.

**Table 1:** Demographic characteristics of the study population.

Clinical Condition	Number of samples	M	F	Mean age ± SD
Barrett’s esophagus	10	6	4	50.6±6.4
Esophageal adenocarcinoma	6	4	2	55.8±3.2
Controls	10	6	4	51.6±7.2

SD: standard deviation.

### Microbiota composition across esophageal disease stages

**Fig 1:**
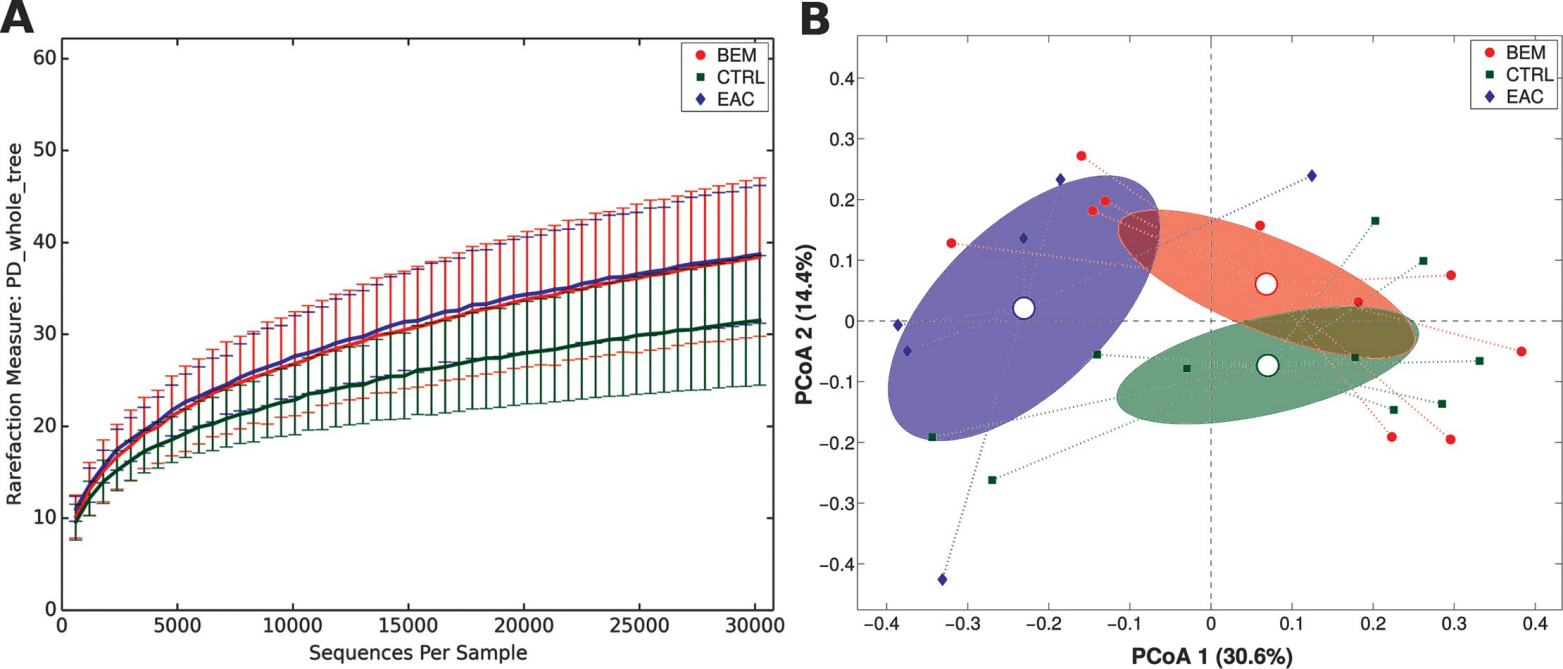
(A) Alpha-diversity rarefaction curve for Faith’s phylogenetic diversity (PD_whole_tree) metric for BEM, EAC or healthy control samples. Curves represent the average value of all the samples within the experimental category; error bars represent standard deviations. (B) PCoA plot of the Bray-Curtis distances among samples; each point represents a sample, centroids are calculated as the mean coordinate of all samples per experimental category (BEM, EAC or CTRL); ellipses represent the SEM-based estimation of the variance. The first and second components of the variance are shown. BEM: esophageal metaplastic samples; EAC: esophageal adenocarcinoma samples; CTRL: healthy control samples.

Gut microbiota biodiversity and composition for each group were analyzed via α- and β-diversity values. BEM and EAC samples showed an overall higher level of α-diversity compared with CTRL, although the difference was not statistically significant (**Fig 1A**).

On the other hand, when evaluating β-diversity, a significant difference was observed between CTRL and EAC using unweighted Unifrac (p = 0.02) and Bray-Curtis distances (p = 0.018), as well as between BEM and EAC at Bray-Curtis evaluation (p = 0.034) (**Fig 1B**). No significant differences were registered considering weighted UniFrac distance.

**Fig 2:**
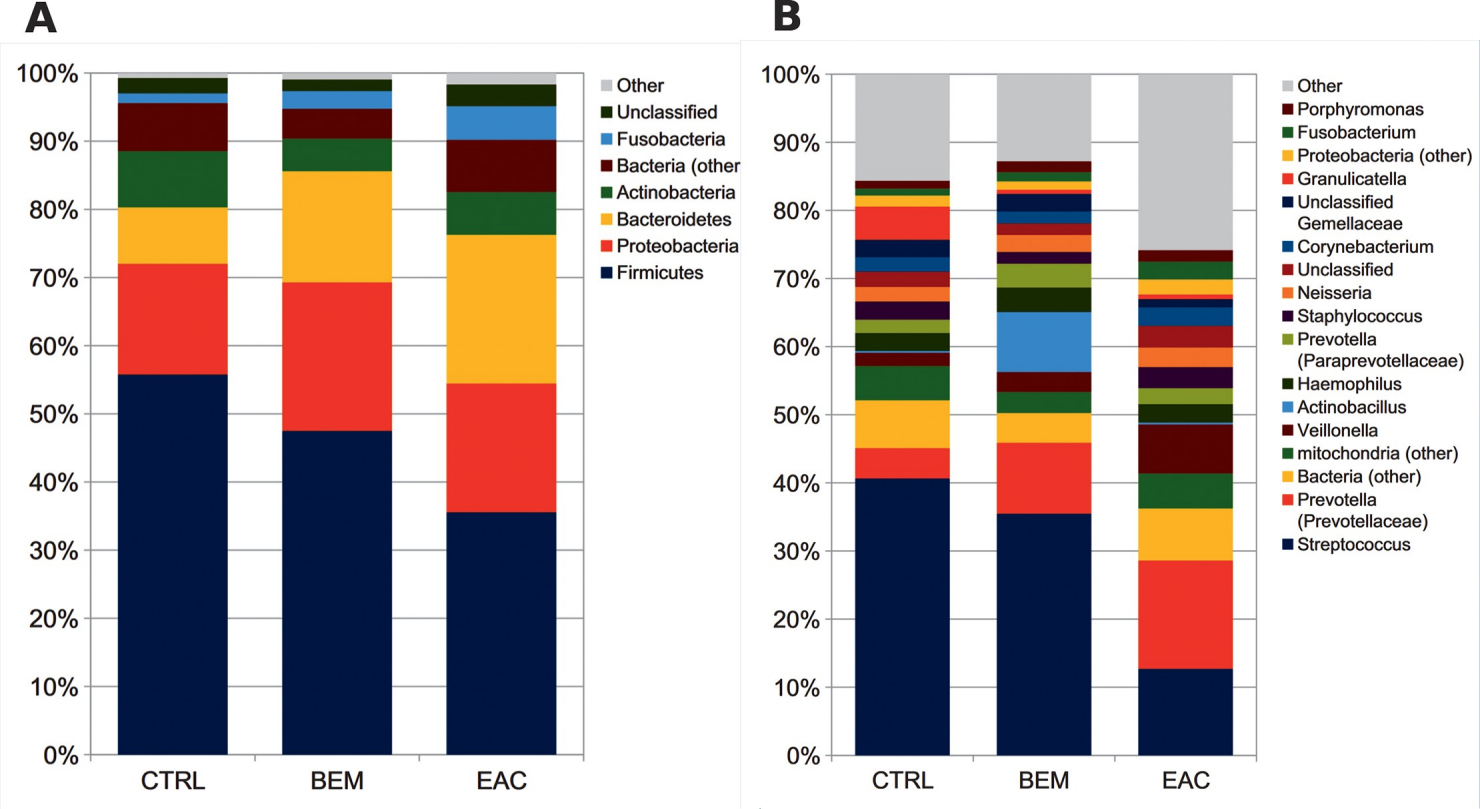
Barplots of the relative abundance of the main bacterial taxa at (A) phylum or (B) genus level for BEM, EAC or healthy control samples. Data represent the average of the relative abundance of all samples per experimental category. Only groups with an average rel. ab ≥1.5% are plotted. BEM: esophageal metaplastic samples; EAC: esophageal adenocarcinoma samples; CTRL: healthy control samples.

In order to reveal distinctive characteristics of each group, taxa distribution was explored at the phylum and genus level. Normal esophageal mucosa was composed mainly by *Firmicutes* (55.7% average relative abundance, rel. ab.), *Proteobacteria* (16.2%), *Bacteroidetes* and *Actinobacteria* (8.2% each), *Fusobacteria* (1.4%), plus another ~7% of unidentified bacteria. At the genus level, *Streptococcus* (40.6% average rel. ab.) was the main contributor to the microbiota profile, followed by *Granulicatella* and *Prevotella* (rel. abs. 4.9% and 4.5%, respectively); other subdominant genera were *Haemophilus*, *Staphylococcus*, *Veillonella*, *Propionibacterium* and *Rothia*, accounting for about 2% each. Notably, we also found about 5% of mitochondrial 16S DNA (**Fig 2A and 2B**).

BEM mucosa showed a tendency towards a decrease of *Streptococcus*, *Granulicatella* and *Propionibacterium* and towards an increase of *Prevotella*, *Actinobacillus* and *Veillonella*, together with a statistically significant (p = 0.007) robust increase of *Leptotrichia* and its corresponding phylum *Fusobacteria* (p = 0.038).

EAC mucosa, on the other hand, displayed profound alterations in its microbial composition, as compared to CTRL samples, such as a striking reduction in *Streptococcus* (12.7% rel. ab., p = 0.016 *vs*. both CTRL and BEM) and *Granulicatella* (0.7%) abundance, with a corresponding increase in *Prevotella* (15.9%, p = 0.031), as well as of the corresponding phylum (*i*.*e*., *Bacteroidetes*, p = 0.031), *Veillonella* (7.2%, p = 0.028), and *Leptotrichia* (2.3%) (**Fig 2A and 2B**). These results were also concordant with those from LefSe analysis, suggesting that the main bacterial taxa distinguishing EAC were *Leptotrichia* (phylum: *Fusobacteria*), members of family *Veillonellaceae*, and *Clostridium* and *Moryella* from family *Lachnospiraceae*. On the other hand, CTRL samples were characterized by an increased abundance of the genus *Bacillus* and *Streptococcus* and their respective families (all within class: *Bacilli*) (see **S1 Fig**).

BEM and EAC mucosa samples were characterized also by a shift in the proportion of the different species belonging to *Veillonella* and *Prevotella* genera, compared with CTRL. In particular, BEM and EAC showed a tendency towards a decrease of *V*. *dispar* and *V*. *tobetsuensis*, and an increase of *V*. *atypica* and of other unclassified members of *Veillonella* genus. Within *Prevotella* genus, evident shifts were registered for *P*. *melaninogenica* (decreased in both EAC and BEM) and the unclassified members of the genus (increased in both EAC and BEM). Moreover, BEM samples were characterized by a higher presence of *P*. *histicola*, whereas EAC seemed to have a slightly higher proportion of *P*. *nigrescens* (see **S2 Fig**).

Taken together, these findings identify peculiar microbial characteristics for each group of samples, which share particular features, but can be differentiated at various levels in terms of phylogenetic diversity and relative abundance of specific phyla and genera.

### Taxonomic co-abundances clusters

**Fig 3:**
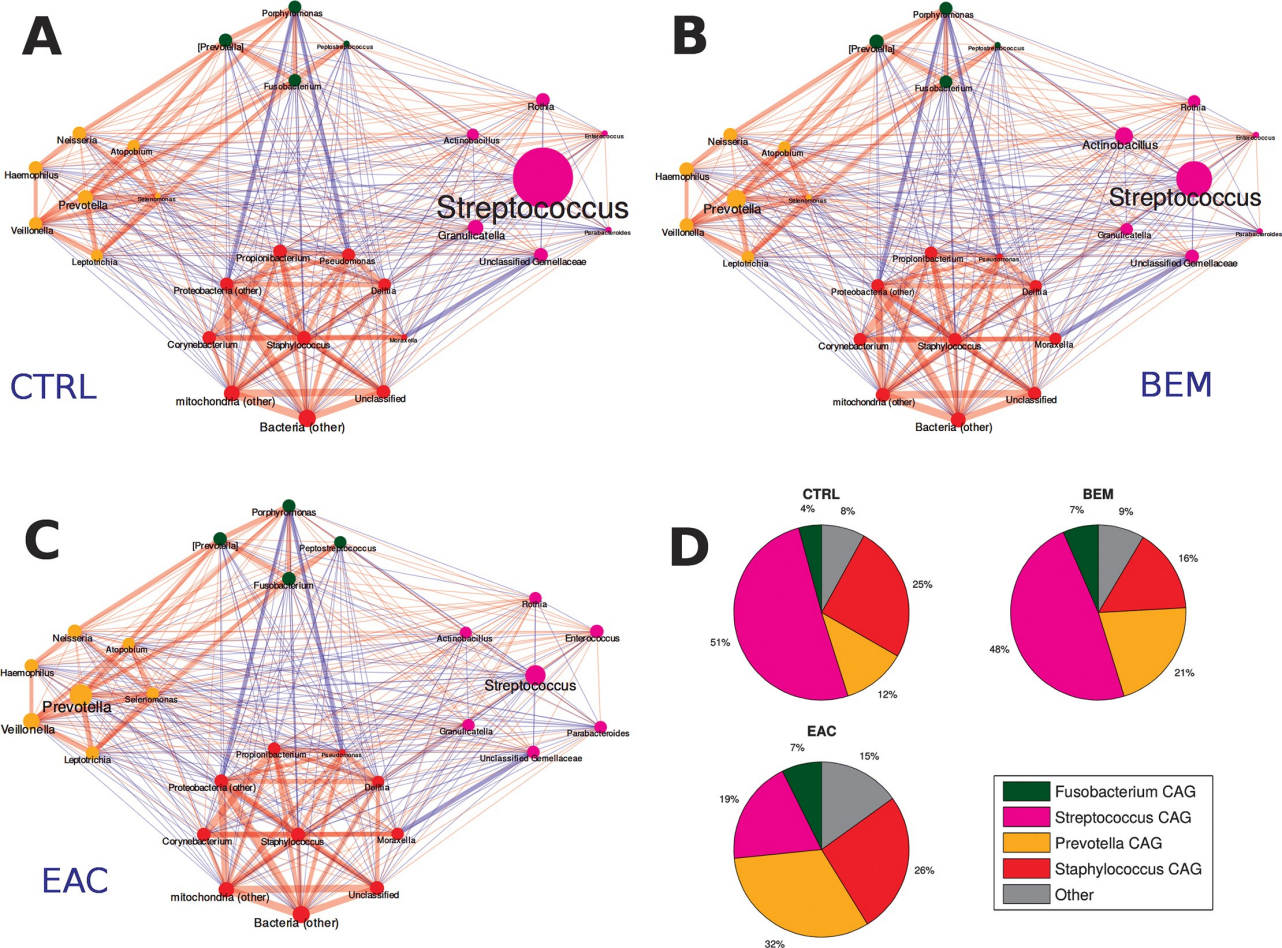
Taxonomic correlations among co-abundant groups (CAGs) in (A) healthy (CTRLS), (B) BEM and (C) EAC individuals. Red edges indicate a positive correlation, while blue edges a negative one. Edge size is proportional to the correlation coefficient. Node and label size represent taxonomy abundance, while the colour indicates the belonging cluster: *Streptococcus* CAG in magenta, *Fusobacterium* CAG in green, *Staphylococcus* CAG in red, and *Prevotella* CAG in yellow. (D) Pie-charts showing the average cumulative relative abundance per CAG and experimental group. CAGs: co-abundant groups; BEM: esophageal metaplastic samples; EAC: esophageal adenocarcinoma samples; CTRL: healthy control samples.

To identify patterns of co-expression among bacterial genera of esophageal microbiota, we determined co-abundances associations on the whole dataset and clustered them into four CAGs, whose names were assigned according to the most abundant or representative genera (**Fig 3A, 3B and 3C**).

Three groups were composed by networks of strongly positively correlated bacteria: *Staphylococcus* CAG (summing up to 21.7% rel. ab. on average) included, among all, *Propionibacterium*, *Pseudomonas* and *Corynebacterium*; *Fusobacterium* CAG (5.8% average rel. ab.), which comprised also *Porphyromonas* and *Peptostreptococcus*; and *Prevotella* CAG (20.1% average rel. ab.), including *Leptotrichia* and *Veillonella* genera. The last CAG (*i*.*e*., *Streptococcus* CAG, accounting for 42.5% rel. ab.) was composed, beside the genus itself, by others, such as *Granulicatella*, *Actinobacillus* and *Parabacteroides*, all showing a substantial non-correlation among themselves or to the other CAGs. Bacterial genera not belonging to the four aforementioned CAGs summed up to 9.9% rel. ab. on average. (see **S3 Fig**).

In control subjects, *Streptococcus* and *Staphylococcus* CAGs dominated the microbiota, summing up to 75.9% of rel. ab., with *Prevotella* and *Fusobacterium* CAGs accounting for 11.8% and 4.0% rel. ab., respectively. BEM group showed a tendency, although not statistically significant, towards the reduction of *Staphylococcus* CAG (15.6% rel. ab.) and the increase in *Prevotella* CAG (21.1% rel. ab.), as well as of its members *Prevotella*, *Veillonella* and *Leptotrichia*. EAC samples were characterized by a marked significantly decrease (p = 0.03) of *Streptococcus* CAG down to 19.3% of rel. ab. and an increase of *Prevotella* CAG (p = 0.04). Notably, this CAG comprised both *Prevotella* and *Veillonella*, which resulted significantly increased compared to CTRL. Finally, EAC group also showed a higher contribution by bacterial taxa not belonging to the four main CAGs as compared to CTRL (15.1% *vs*. 8.1%, p = 0.03) (**Fig 3D**).

### Differences in the microbiota of Barrett patients’ metaplastic and unaffected mucosa

**Fig 4:**
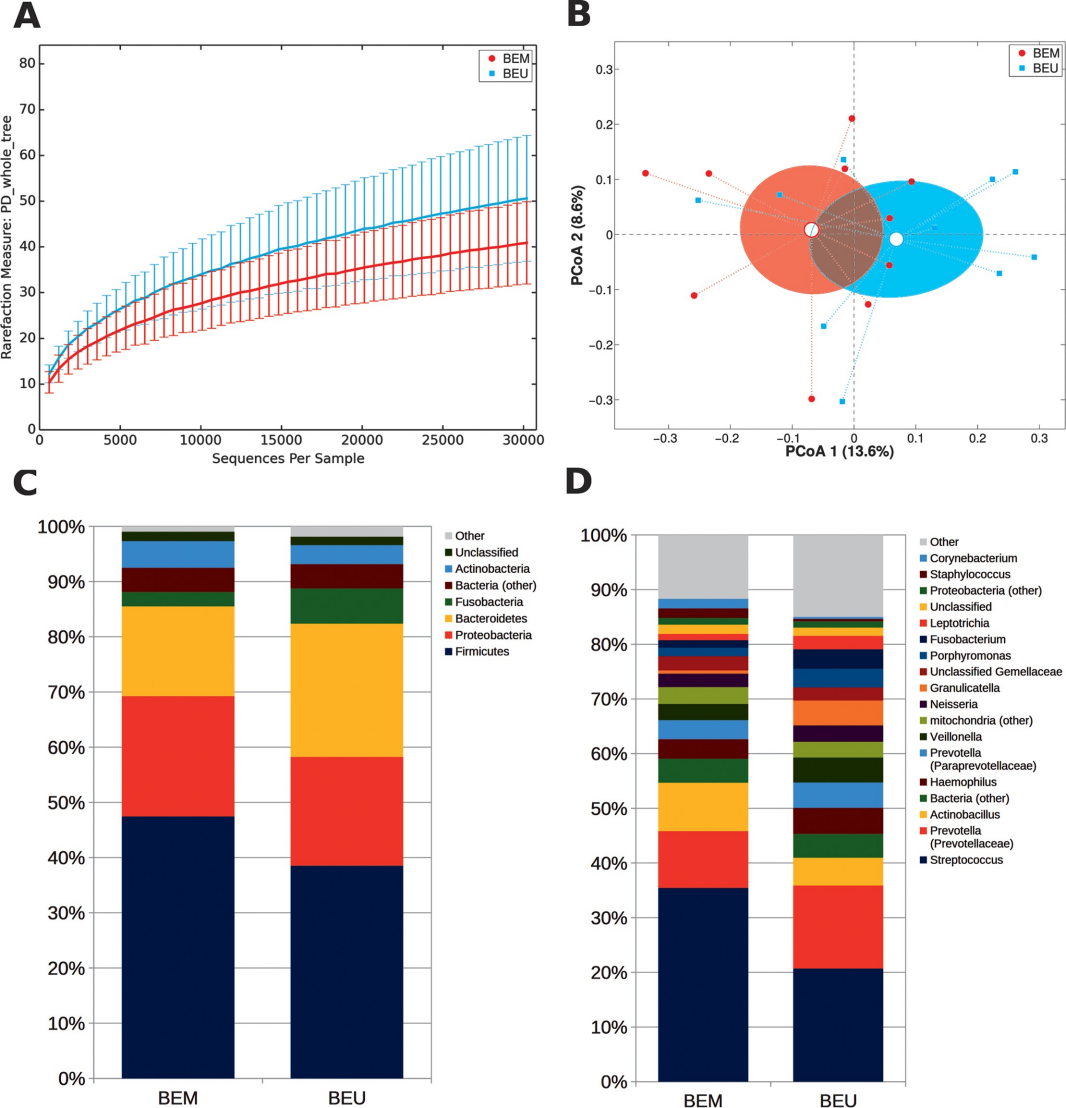
(A) Alpha-diversity rarefaction curve for Faith’s phylogenetic diversity (PD_whole_tree) metric for BEM and BEU samples from BE patients. Curves represent the average value of all the samples within the experimental category; error bars represent standard deviations. (B) PCoA plot of the Bray-Curtis distances among samples; each point represents a sample, centroids are calculated as the mean coordinate of all samples per experimental category (BEM or BEU); ellipses represent the SEM-based estimation of the variance. The first and second components of the variance are shown. (C,D) Barplots of the relative abundance of the main bacterial taxa at phylum or genus level for BEM or BEU samples. Data represent the average of the relative abundance of all samples per experimental category. Only the 7 most abundant phyla and the 20 most abundant genera are plotted. BEM: esophageal metaplastic samples; BEU: normal esophageal samples obtained from patients with Barrett’s esophagus.

We further explored if microbiota compositional differences may reflect the division between metaplastic and adjacent healthy tissue. This resulted in a trend towards a decreased α-diversity in BEM areas (p = 0.102, PD_whole_tree metric) and a close clustering between BEM and BEU samples (**Fig 4A and 4B)**. However, metaplastic tissue showed a reduced abundance of several groups, compared to adjacent healthy areas, including: *Bacteroidetes* (p = 0.049) and *TM7* (p = 0.002) at the phylum level; *Prevotellaceae* (p = 0.027), *Veillonellaceae* (p = 0.014), *Fusobacteriaceae* (p = 0.027), *Lachnospiraceae* (p = 0.048) and *Campylobacteraceae* (p = 0.008) at the family level; and *Prevotella* (p = 0.027), *Fusobacterium* (p = 0.04), *Campylobacter* (p = 0.008) and *Selenomonas* (p = 0.006) at the genus level (**Fig 4C and 4D)**. As expected, phylogenetic distances were more similar between samples from the same patient than across different individuals (p<0.05 for all distances, see **S4 Fig**).

### Microbiota-related functional profiles across esophageal disease stages

**Fig 5:**
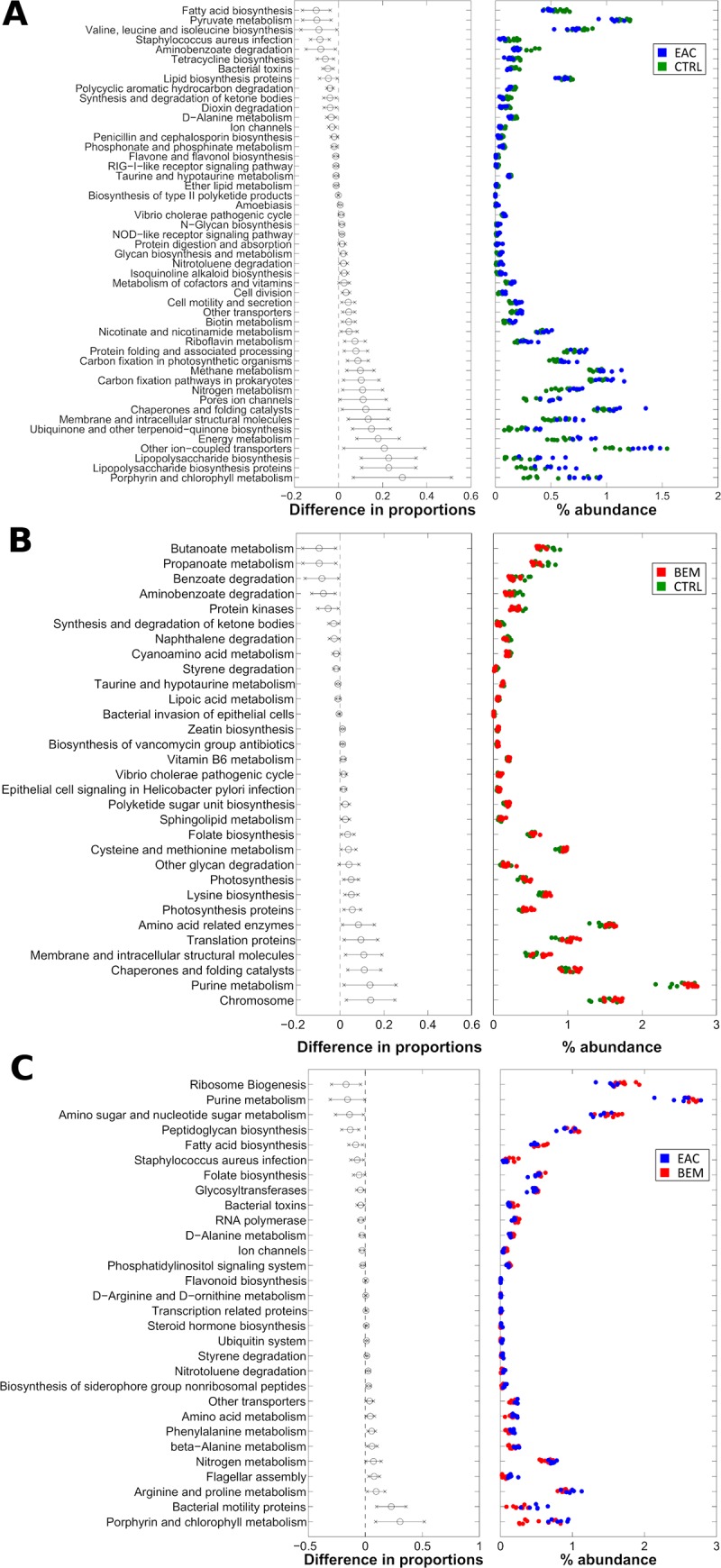
Level-3 KEGG pathways significantly different between (A) EAC and CTRL, (B) BEM and CTRL (C) EAC and BEM samples from PiCRUST functional prediction. Pathways are represented on the basis of increasing difference between average proportions in the experimental categories (Black circles, leftmost panel); for each pathway, the 95% confidence interval is also represented. Rightmost panel shows the % abundance of each sample; colors indicate the experimental categories. BEM: esophageal metaplastic samples; EAC: esophageal adenocarcinoma samples; CTRL: healthy control samples.

We finally investigated microbiota-related functional predictions of EAC and BEM compared to those of healthy controls (**Fig 5**). Patients with EAC showed a significant upregulation of microbial genes related to energy metabolism, metabolism of cofactors and vitamins, cellular processes and signaling, while fatty-acids biosynthesis and nitrogen and D-alanine pathways were consistently down-regulated compared to CTRL (**Fig 5A**). Microbiota associated to BEM was characterized by a higher potential for replication and repair, genetic information processing, metabolism of cofactors and vitamins, energy metabolism, amino acids, nucleotides, lipids and glycan metabolisms, while showed robustly reduced xenobiotics biodegradation and metabolism, carbohydrates and lipoic acid metabolism compared with control-associated microbiota (**Fig 5B**).

Finally, when comparing EAC and BEM samples, EAC-associated microbiota showed a significantly increased amino acid metabolism, energy metabolism, porphyrin, beta-alanin, D-arginine and D-ornithine metabolism. Conversely, it displayed a consistently decreased, carbohydrate metabolism, glycan biosynthesis and metabolism, folate biosynthesis and nucleotide metabolism pathways (**Fig 5C**).

### Microbiota-related functional profiles in Barrett patients’ metaplastic and unaffected mucosa

**Fig 6:**
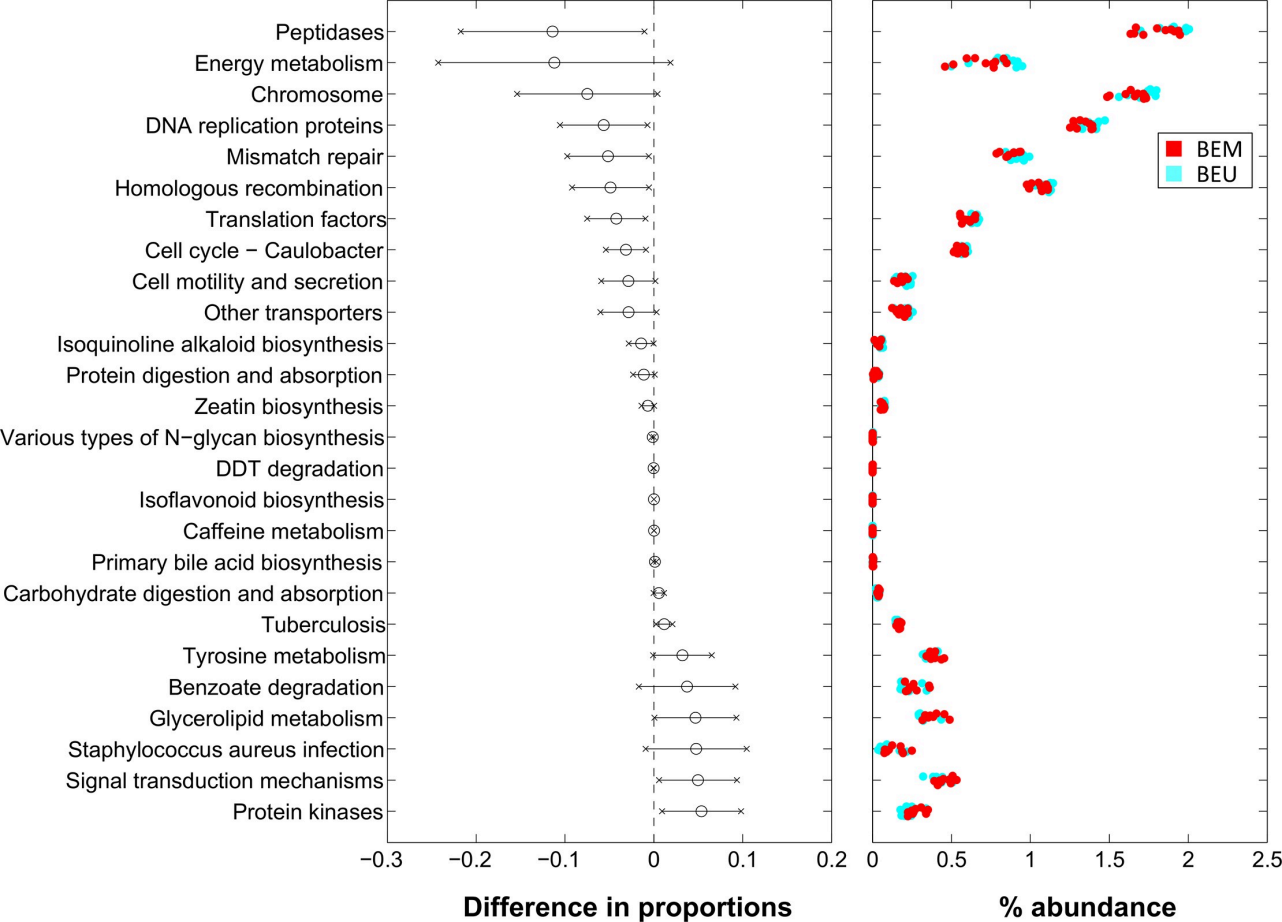
Level-3 KEGG pathways significantly different between BEM and BEU samples from PiCRUST functional prediction. Pathways are represented on the basis of increasing difference between average proportions in the experimental categories (Black circles, leftmost panel); for each pathway, the 95% confidence interval is also represented. Rightmost panel shows the % abundance of each sample; colors indicate the experimental categories. BEM: esophageal metaplastic samples; BEU: normal esophageal samples obtained from patients with Barrett’s esophagus.

Differences in the microbial composition reflected also in a different predicted functional profile between BEM and BEU samples coming from the same individual (**Fig 6**). In particular, BEM mucosa samples showed a significant depletion of pathways belonging to replication and repair and translation (*i*.*e*., chromosome, DNA replication proteins, mismatch repair, homologous recombination and translation factors), while DDT degradation, benzoate degradation (belonging to xenobiotics degradation and metabolism L2-pathway), primary bile acid biosynthesis and glycerolipid metabolism (belonging to lipid metabolism L2-pathway) were increased. Other notable differences involved enzyme families, with peptidases decreased and protein kinases increased in BEM as compared to BEU. Finally, isoflavonoid biosynthesis, caffeine metabolism and tyrosine metabolism were augmented in BEM, whereas isoquinoline alkaloid biosynthesis, N-glycan biosynthesis and zeatin biosynthesis were depleted.

## Discussion

Increasing evidence has supported a critical role of microbiota in GI [5–8] and extra-intestinal diseases [9–12], and also in various types of cancers [13, 14]. However, few information exist on upper GI, while the majority of studies have focused on sites easier and less invasive to sample. Moreover, the available studies have not been designed to comprehensively evaluate through 16S-based amplicon sequencing the whole normal-BE-EAC human mucosal spectrum and thus have yielded mixed results [24, 39, 40]. In the present study, we report not only a thorough characterization of the microbial signature that differentiates BE and EAC, but also underline the microbial regional differences between BEU and BEM mucosa. Firstly, we found that a higher number of OTUs distinguishes BE and EAC from control samples. Indeed, α-biodiversity analysis showed higher values for BE and EAC, although the difference was not statistically significant. β-diversity assessment suggested a trend towards a clusterization of three groups with a different phylogenetic composition between controls and EAC, as well as between BEM and EAC. This indicated a potential relationship between the disease and the local microbial community. In order to reinforce this hypothesis, we, then, explored taxonomic characteristics for each group. At the phylum level, a progressive reduction of *Firmicutes*:*Bacteroidetes* ratio was registered from BE to EAC. Members of the phylum *Firmicutes* are known to be the major components of the microbiota of the normal esophagus [24]. Of note, this is in contrast with the composition of intestinal microbiota where *Bacteroidetes* represent the predominant phylum and a dysbiotic increase of *Firmicutes*:*Bacteroidetes* ratio is correlated with intestinal and extra-intestinal diseases [41]. *Firmicutes*:*Bacteroidetes* ratio reduction was mainly explained by a progressive reduction of *Streptococcus* relative abundance followed by a corresponding increase of *Prevotella* in BEM, which appeared even more marked and significant in EAC. A co-exclusion association between the two dominant taxa *Streptococcus* and *Prevotella* is constantly detected in the studies on esophaegeal microbiome [23]. A previous report on reflux esophagitis and BE characterized distal esophagus microbiota into two types. Type I microbiome was correlated with normal esophagus and, mainly, contained Gram-positive bacteria, primarily *Firmicutes*, and especially *Streptococcus*. Type II microbiome, mainly associated with reflux esophagitis and BE, was predominated by Gram-negative anaerobes and microaerophiles (phyla *Bacteroidetes*, *Proteobacteria*, *Fusobacteria*, and *Spirochaetes*) [41]. Further studies established that the dominant taxon within the healthy esophagus is *Streptococcus* [42] and that the EAC cascade is depicted by a shift towards a prevalence of Gram-negative species [22]. While following analyses have not replicated these observations [43], others have included the increase enrichment of specific Gram-negative species such as *Campylobacter* and *Fusobacterium* in the EAC process [44, 45]. Our data confirm the characteristics of normal microbiota at esophageal level, with *Streptococcus* representing the main component. Beside this, it portrays EAC as an extreme dysbiotic perturbation of BEM microbiota, which is dominated by Gram-negative anaerobes. Among these, *Leptotrichia* (phylum: *Fusobacteria*), despite being a subdominant member of the community, emerges as the principal discriminating genus for EAC. *Fusobacteria* are anaerobic, non-spore-forming, Gram-negative bacilli with two major families, *Leptotrichiaceae* and *Fusobacteriaceae* usually identified in the human oral cavity [46]. Although a broad genetic diversity characterizes *Leptotrichia*, with six species belonging to this genus identified so far [47], its exact involvement in human physiology is still not well established. It is considered an opportunistic pathogen, which is more prone to trigger disease concomitantly with local or systemic predisposing conditions. It has been described in several human infections [47–49] and human cancers, such as colon [50], gastric [51] and pancreatic neoplasia [52]. *Leptotrichia* stimulates a human immune response and serum anti-*Leptotrichia* antibodies are common [47]. We can speculate that the immune activity provoked by *Leptotrichia*, rather than *Leptotrichia* itself, may facilitate esophageal tumorigenesis.

Moreover, *Leptotrichia* increase in BEM and EAC is followed by a corresponding elevation of *Veillonella* and *Prevotella*, other bacteria commonly found in the oral cavity, which could be plausible pro-oncogenic partners, as they have been reported to be associated with different cancers [51, 53–57]. The enrichment of oral organisms is increasingly being shown across several types of tumor [58, 59]. This is in line with the theory that EAC cascade may be linked to an enrichment of a diverse group of microorganisms with common features rather than specific microorganisms [23].

Interestingly, these genera are forming a network of positively correlated bacteria (*Prevotella* CAG) strictly linked to BEM and EAC. Their growth is balanced by a corresponding significant reduction of clusters linked to the healthy condition. Indeed, BEM group showed a tendency, although not statistically significant, towards the reduction of *Staphylococcus* CAG and an increase of *Prevotella* CAG. EAC samples were characterized by a marked decrease of *Streptococcus* CAG and an increase of *Prevotella* CAG. The enrichment of networks of oral organisms has been reported in gastric cancer [51] and colorectal cancer [60] and it has been associated with inflammation in the gut [61].

The variation of these main clusters could sustain crucial physio-pathological consequences at mucosal level and promote a pro-inflammatory environment. Firstly, we can hypothesize that the increase and co-aggregation of Gram-negative organisms could lead to the formation of a biofilm and to the activation of the innate immune response through specific molecules, such as lipopolysaccharide (LPS) [41], with consequent stimulation of the nuclear factor kappa-B (NF-kB) expression and the release of inflammatory cytokines (*i*.*e*., interleukin-1β, -6, -8, and tumor necrosis factor-α). At the same time, *Bacteroidetes* could contribute to the maintenance of other microbial members by providing essential factors such as short fatty acids and the CO_2_ and H_2_ gas [22]. This could sustain the inflammatory and tumorigenic process along the BE-EAC spectrum [21, 62, 63]. Indeed, our predictive functional analysis confirmed this hypothesis and also pointed out peculiar functional profiles for each group of samples. Microbiota associated to BEM was characterized by a high potential for replication and repair. Similarly, patients with EAC showed significantly upregulated microbial genes related to energy metabolism, replication and signaling, with, interestingly, the fatty-acids biosynthesis and nitrogen and D-alanine pathways consistently down-regulated, also when compared with BEM.

Finally, we explored potential regional microbiota variations among BE samples by distinguishing the metaplastic areas from those with normal mucosa. Our data underlined not only a clearly different microbial structure between BEM and BEU, but also an unexpected important distinction at the microbiological and inferred functional level between the normal mucosa obtained from BE patients and that biopsied from controls, reinforcing the innovative concept that microbiota could represent a predisposing factor of BE. At the same time, this distinction could be due to a potential influence exerted on BEU by the microbiota inhabiting the near metaplastic areas.

Overall, our study describes peculiar microbial compositional and functional differences using 16S-based amplicon sequencing to compare normal, BEU, BEM and EAC mucosa in humans. This study is not without limitations. Despite the significant alterations identified, this is a pilot study with a low number of patients. Additional studies with a larger cohort of subjects are warranted. However, although the number of mucosal samples collected was small, patients’ selection and sample processing were conducted in order to reduce confounding factors.

Other studies have indicated that genetic tests could characterize BE and EAC and predict the disease progression [64, 65]. Our data show that specific microbial markers can, further, differentiate BEU, BEM and EAC. The identification of the microbial communities associated with cancer is of crucial importance in order to find risk factors and to hypothetically guide surveillance protocols. Thus, if coupled, genetic and microbial markers may help to prevent EAC or detect it at earlier, treatable stages, reducing the need for repeated surveillance procedures on large number of patients who never progress to cancer, thus ameliorating the management of this debilitating disease.

## Supporting information

S1 File (PDF)

S1 Fig Cladogram from LefSE (Linear discriminant analysis Effect Size) analysis on EAC, BEM and healthy controls. Cladogram shows taxa likely to distinguish experimental classes. Phylum, classes and orders are reported on the cladogram, whereas differential families and genera are named in the legend. No feature was found for BEM samples. BEM: esophageal metaplastic samples; EAC: esophageal adenocarcinoma samples; CTRL: healthy control samples.
(TIF)

S2 Fig Boxplots showing the relative abundance of some (A) Veillonella and (B) Prevotella species. The contribution of each species is reported as a proportion on the total reads in the corresponding genus. Only samples with ≥0.5% rel. ab. in the specific genus were considered. Each point represents a sample; median values are reported as yellow lines, whereas means are in cyan. BEM: esophageal metaplastic samples; EAC: esophageal adenocarcinoma samples; CTRL: healthy control samples.
(TIF)

S3 Fig Definition of bacterial co-abundance groups (CAGs). (A) Heatmap used to define CAGs, showing the Kendall correlation coefficient between genera and hierarchically clustered on the basis of Euclidean distance and Ward linkage. Only genera present at least at 1% relative abundance in at least 30% of the samples per experimental condition (*i*.*e*., CTRL, BEM, EAC) are shown. Clustering is performed only on genera whose correlation is statistically different from 0 (p*-*value of the linear model <0.05). (B) Network plot highlighting correlation relationships of CAGs for the whole dataset (n = 26). Circle sizes indicate genus abundances and line thickness is proportional to correlation value. Red lines indicate a positive correlation value; blue lines a negative one. (C) Bar plots showing the average cumulative relative abundance of each CAG in the microbiota of the subjects for each experimental group. In grey, the portion of genera not belonging to the identified CAGs due to the initial filtering is represented. BEM: esophageal metaplastic samples; EAC: esophageal adenocarcinoma samples; CTRL: healthy control samples.
(TIF)

S4 Fig Analysis of distances between paired BEM and BEU samples. (A) PCoA plot based on the Bray-Curtis distances among samples. Paired data from BEU and BEM mucosal biopsies from the same BE patient (n = 10) are shown. (B) Boxplot of intra- (within) and inter-sample (between) distances. “Within” are the distances between each paired BEM-BEU sample from the same patient; “Between” samples distances are calculated as the median of all the distances between each BEM sample and the BEU sample from other patients. BEM: esophageal metaplastic samples; BEU: normal esophageal samples obtained from patients with Barrett’s esophagus.
(TIF)

S5 Fig Boxplots showing the relative abundance of the main (A) phyla and (B) genera in BEM, EAC and CTRL samples. Each point represents a sample; median values are reported as yellow lines, whereas means are in cyan. BEM: esophageal metaplastic samples; EAC: esophageal adenocarcinoma samples; CTRL: healthy control samples.
(TIFF)

S6 Fig (TIFF)
