# Supplementary material for: Esophageal microbiome signature in patients with Barrett’s esophagus and esophageal adenocarcinoma
Source: PLoS One. 2020 May 5;15(5):e0231789. doi: 10.1371/journal.pone.0231789 (PMC7199943; doi:10.1371/journal.pone.0231789)
Supplement: S1 File — (PDF) [file pone.0231789.s001.pdf]

SUPPLEMENTAL INFORMATION APPENDIX

Supplemental Material and Methods

Species-level analysis

Classification of reads belonging to the main bacterial genera of the collected samples was further improved, where possible, down to the species level, via a BLAST-based re-classification on an *ad-hoc* built reference database, based on the sequences available from NCBI RefSeq database for bacteria (ftp://ftp.ncbi.nlm.nih.gov/genomes/refseq/bacteria/), which comprised, as of 2018, February 12th a total of 17898 species, belonging to 2259 genera.

Reference sequences

Through a custom script, for each genus, sequenced genome for all species and strains were downloaded and properly formatted for further processing. In all our analyses, only bacterial strains with a genome finishing grade of “Complete”, “Chromosome” or “Scaffolds” were considered. The following table summarizes the references used for each of the groups considered.

Genus name	Number of species	Number of strains
Streptococcus	178	6610
Prevotella	77	146
Veillonella	15	25
Leptotrichia	12	15

Reads to re-classify

From the OTU table comprising all the samples, OTUs classified within the above genera were

selected and the sequences of all the reads grouped in each OTU (clustered at 97% similarity) were retrieved. In order to reduce the number of sequences to re-classify, clonal reads (i.e.: reads being identical throughout 100% of their length and composition) were grouped together.

Classification

Re-classification of the reads was performed through nucleotide BLAST (legacy BLAST, v 2.26) [1], using a cutoff of 1e-10 for the e-value and de-activating the dust-filter. Only reads matching for at least of 80% of their length were retained and, for each read, the best match (i.e.: that or those with the higher bit-score) was selected. If a read had multiple classifications on different species, the classification was reset to genus level.

Statistical analysis

In order to keep only consistent data for species-level evaluations, only samples having an incidence (i.e.: a relative abundance) higher than 0.5%, were considered. This was made to exclude samples with very few reads classified in the genus that could profoundly alter the dataset (e.g.: considering a sample in which we had only 1 read in a genus, this would have brought a 100% to the species-level classification for that certain species). Since the least sequenced sample had about 12000 reads, this equaled having at least 150 reads in the genus. The following table summarizes the number of samples per each group that were kept:

**Taxa**	**CTRL**	**BEM**	**EAC**
Streptococcus	10	10	6
Prevotella	10	10	5

Veillonella	10	8	5
Leptotrichia	1	5	3

A non-parametric Mann-Whitney U-test was used to compare the relative abundance of each bacterial species in the different experimental groups, considering p-values <0.05 as significant.

Co-abundance network analysis

Bacterial genera were selected considering only those present at >0.5% of abundance in at least 30% of the samples in at least one experimental group, in order to exclude minor and transient contributors of the gut microbiota. This resulted in a subset of 28 genera using the whole dataset, having a relative across all samples ranging from 32.20% to 0.23%.

All statistical evaluations and heatmaps were carried out using Matlab (v 2008b, Natick, MA, USA) and the Fathom Toolbox [2] and visualized by Cytoscape (v 3.0) [3].

The co-abundance between each pair of genera was evaluated calculating the Kendall's correlation coefficient and displayed as heatmaps, hierarchically clustered using Spearman's correlation metric and Ward linkage. Only associations having a Benjamini-Hochberg adjusted p-value <0.05 for the linear model were used to build for the hierarchical clustering. Results obtained considering the entire dataset of samples were used to define the co-abundance groups (CAGs). Permutational multivariate analysis of variance (P-MANOVA) [4] was used to determine whether CAGs were significantly different from each other. Essentially this compared strength of the correlations between the groups to correlation strengths within the groups in a pairwise manner. All comparisons, performed via 9999 random permutations, had a p-value<0.003 for rejecting the hyphothesis of no-difference among groups. Co-abundance network plots were created as previously described [5]. In the plots, circle and label size is proportional to the genus' relative abundance in the experimetal group or along

the whole dataset; circle colors represent the CAGs clusters; red edges suggest a positive correlation between genera, whereas blue edges represent negative correlation; edge thickness is proportional to the Kendall's correlation coefficient.

References

1. Altschul SF, Gish W, Miller W, Myers EW, Lipman DJ. Basic local alignment search tool. J Mol Biol. 1990;215(3):403-10. doi: 10.1016/S0022-2836(05)80360-2. PubMed PMID: 2231712.

2. Jones DL. Fathom Toolbox for Matlab: software for multivariate ecological and oceanographic data analysis.2015. Available from: http://www.marine.usf.edu/user/djones/matlab/matlab.html.

3. Shannon P, Markiel A, Ozier O, Baliga NS, Wang JT, Ramage D, et al. Cytoscape: a software environment for integrated models of biomolecular interaction networks. Genome research. 2003;13(11):2498-504. doi: 10.1101/gr.1239303. PubMed PMID: 14597658; PubMed Central PMCID: PMCPMC403769.

4. Anderson MJ. A new method for non-parametric multivariate analysis of variance. Austral Ecology. 2001;26:32-46.

5. Claesson MJ, Jeffery IB, Conde S, Power SE, O'Connor EM, Cusack S, et al. Gut microbiota composition correlates with diet and health in the elderly. Nature. 2012;488(7410):178-84. doi: 10.1038/nature11319. PubMed PMID: 22797518.
