# Supplementary figures and images for: Esophageal microbiome signature in patients with Barrett’s esophagus and esophageal adenocarcinoma
Source: PLoS One. 2020 May 5;15(5):e0231789. doi: 10.1371/journal.pone.0231789 (PMC7199943; doi:10.1371/journal.pone.0231789)

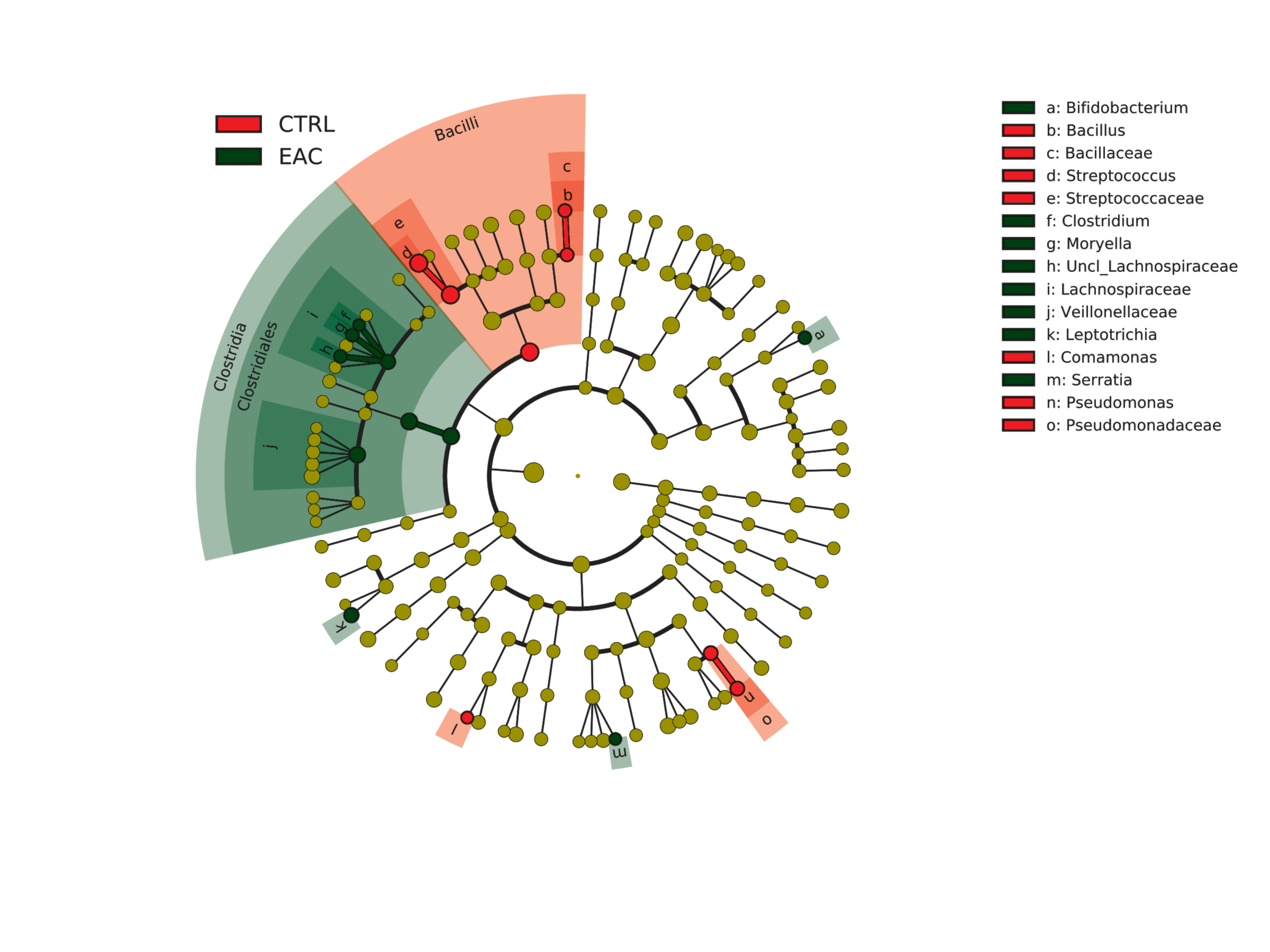

Supplement: S1 Fig — Cladogram shows taxa likely to distinguish experimental classes. Phylum, classes and orders are reported on the cladogram, whereas differential families and genera are named in the legend. No feature was found for BEM samples. BEM: esophageal metaplastic samples; EAC: esophageal adenocarcinoma samples; CTRL: healthy control samples. (TIF) [file pone.0231789.s002.tif]

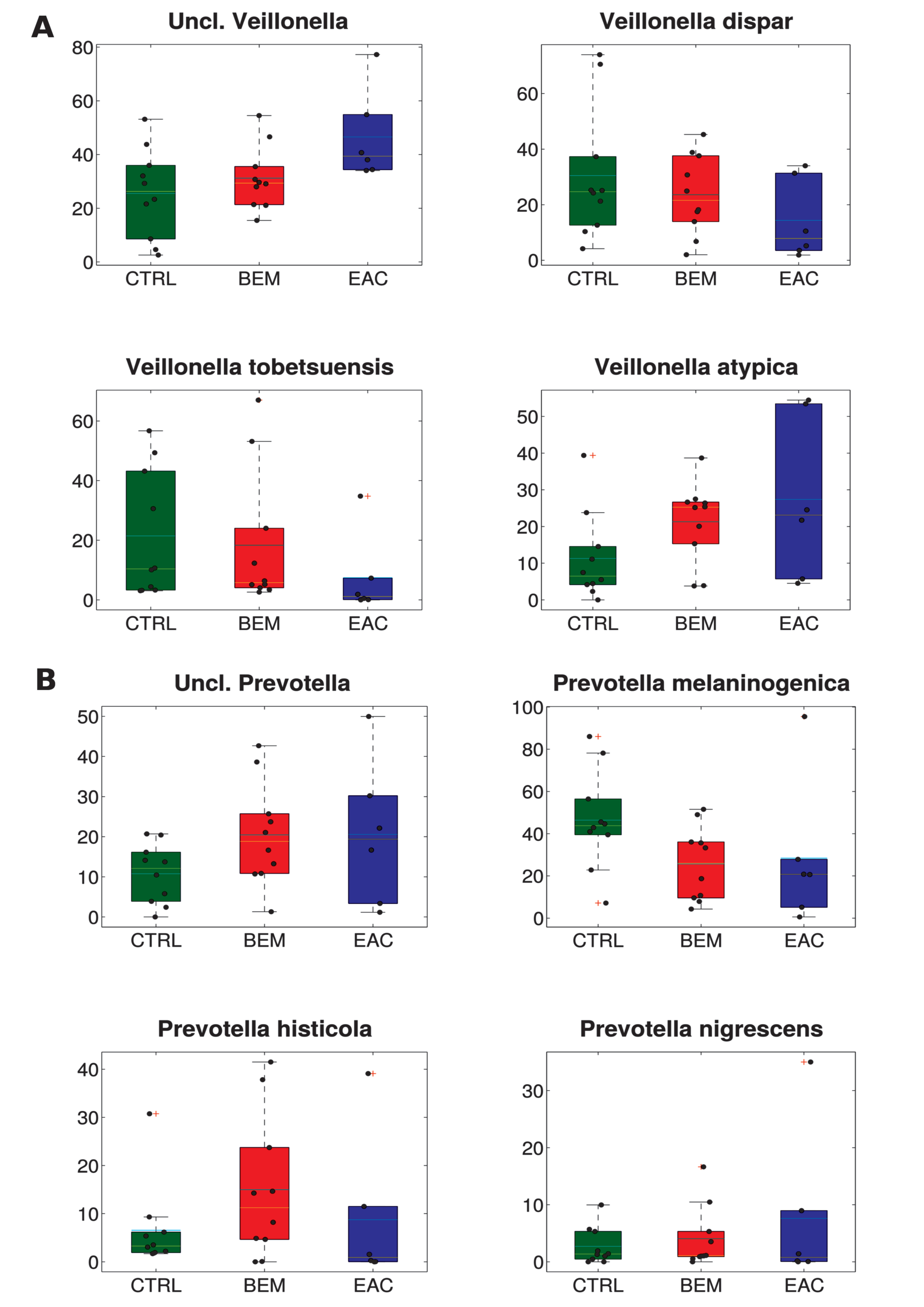

Supplement: S2 Fig — Boxplots showing the relative abundance of some (A) Veillonella and (B) Prevotella species. The contribution of each species is reported as a proportion on the total reads in the corresponding genus. Only samples with ≥0.5% rel. ab. in the specific genus were considered. Each point represents a sample; median values are reported as yellow lines, whereas means are in cyan. BEM: esophageal metaplastic samples; EAC: esophageal adenocarcinoma samples; CTRL: healthy control samples. (TIF) [file pone.0231789.s003.tif]

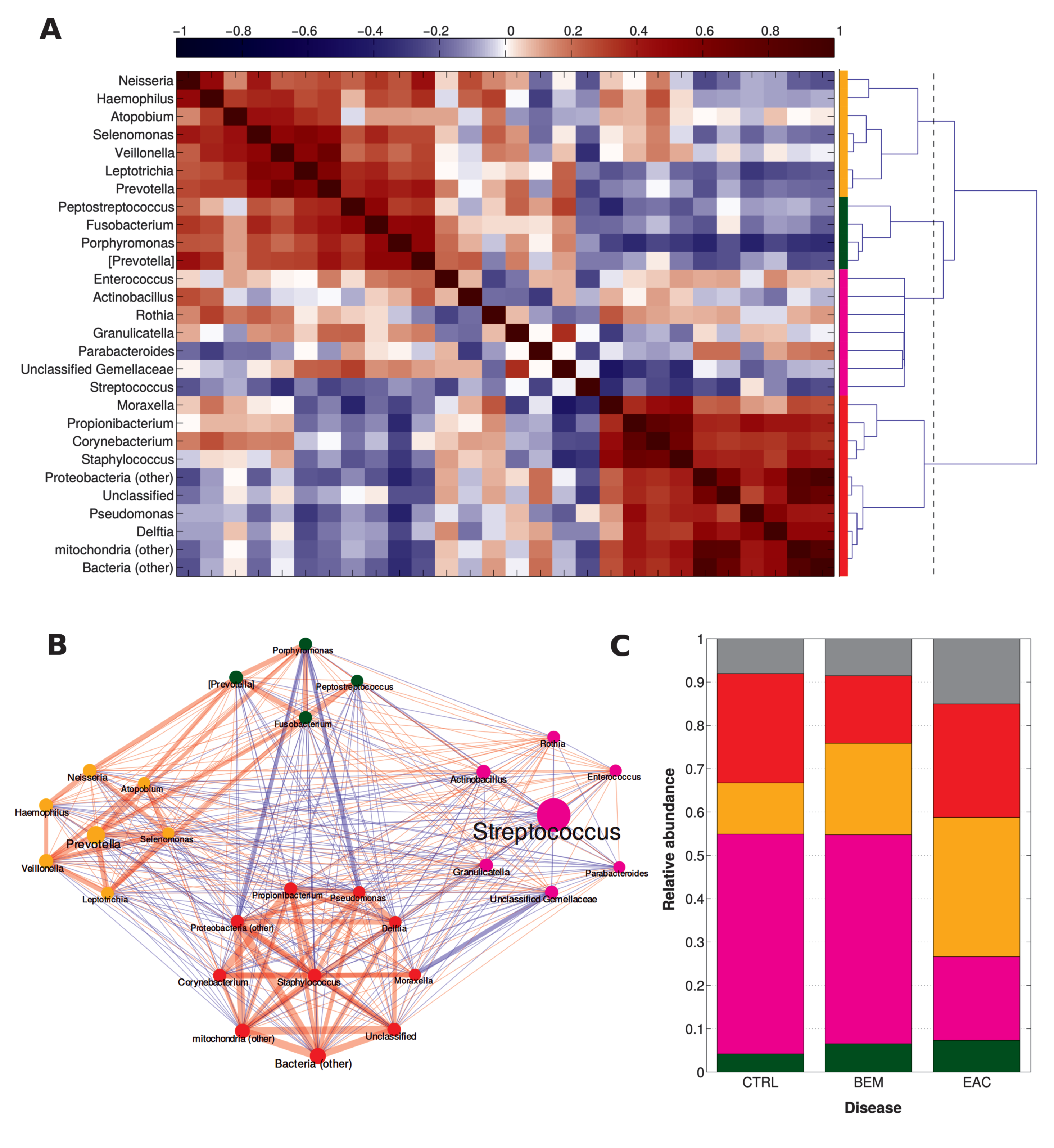

Supplement: S3 Fig — (A) Heatmap used to define CAGs, showing the Kendall correlation coefficient between genera and hierarchically clustered on the basis of Euclidean distance and Ward linkage. Only genera present at least at 1% relative abundance in at least 30% of the samples per experimental condition (i.e., CTRL, BEM, EAC) are shown. Clustering is performed only on genera whose correlation is statistically different from 0 (p-value of the linear model <0.05). (B) Network plot highlighting correlation relationships of CAGs for the whole dataset (n = 26). Circle sizes indicate genus abundances and line thickness is proportional to correlation value. Red lines indicate a positive correlation value; blue lines a negative one. (C) Bar plots showing the average cumulative relative abundance of each CAG in the microbiota of the subjects for each experimental group. In grey, the portion of genera not belonging to the identified CAGs due to the initial filtering is represented. BEM: esophageal metaplastic samples; EAC: esophageal adenocarcinoma samples; CTRL: healthy control samples. (TIF) [file pone.0231789.s004.tif]

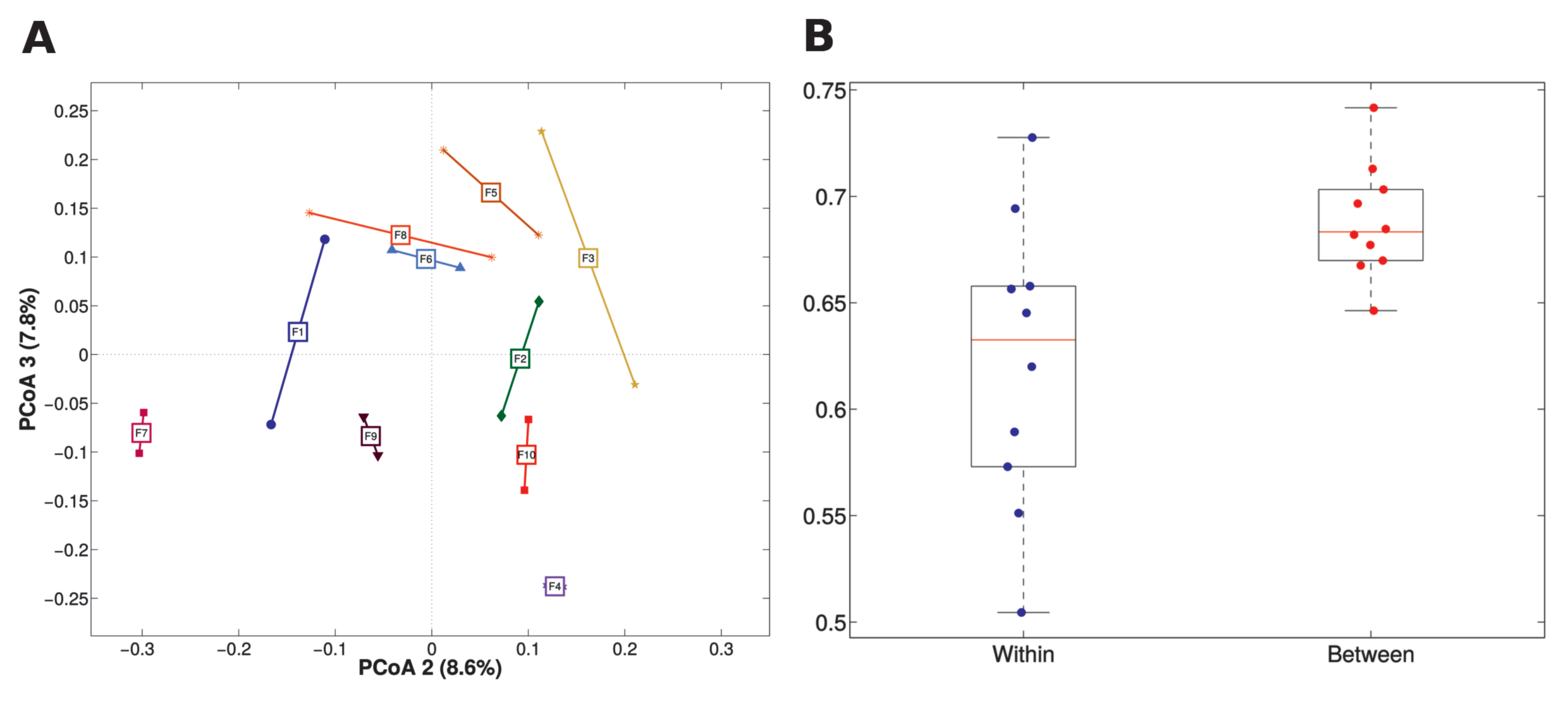

Supplement: S4 Fig — (A) PCoA plot based on the Bray-Curtis distances among samples. Paired data from BEU and BEM mucosal biopsies from the same BE patient (n = 10) are shown. (B) Boxplot of intra- (within) and inter-sample (between) distances. “Within” are the distances between each paired BEM-BEU sample from the same patient; “Between” samples distances are calculated as the median of all the distances between each BEM sample and the BEU sample from other patients. BEM: esophageal metaplastic samples; BEU: normal esophageal samples obtained from patients with Barrett’s esophagus. (TIF) [file pone.0231789.s005.tif]

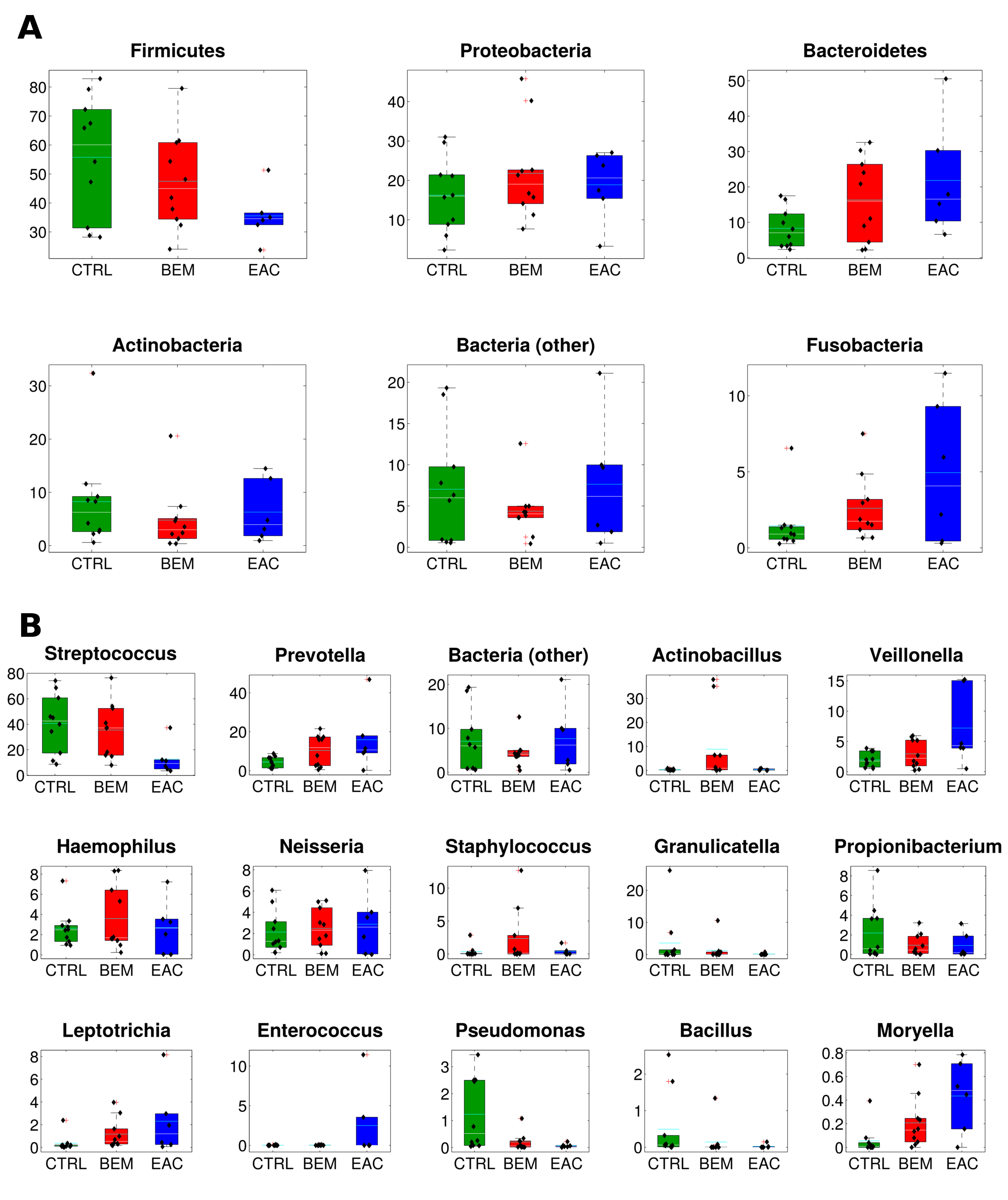

Supplement: S5 Fig — Boxplots showing the relative abundance of the main (A) phyla and (B) genera in BEM, EAC and CTRL samples. Each point represents a sample; median values are reported as yellow lines, whereas means are in cyan. BEM: esophageal metaplastic samples; EAC: esophageal adenocarcinoma samples; CTRL: healthy control samples. (TIFF) [file pone.0231789.s006.tiff]

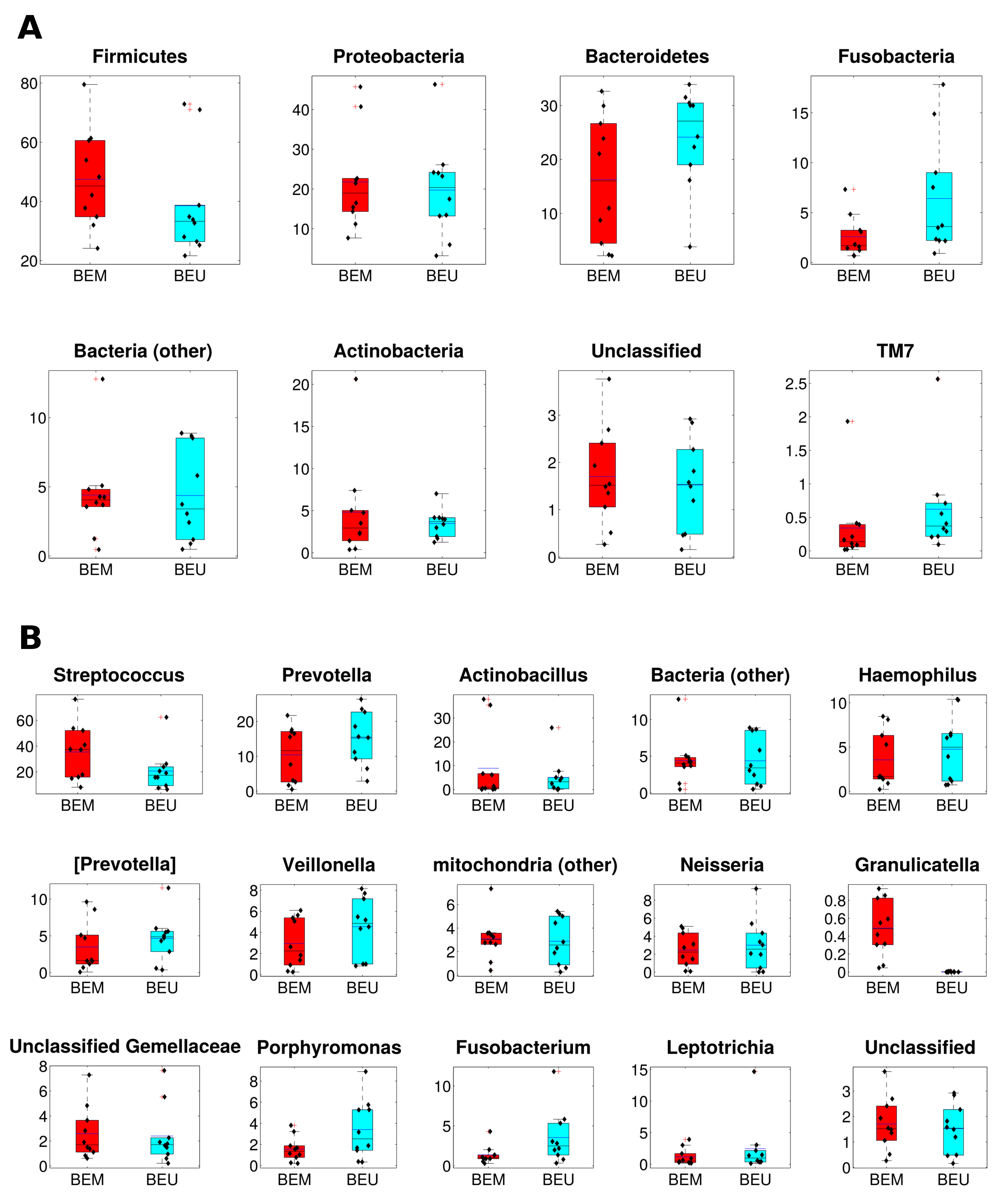

Supplement: S6 Fig — (TIFF) [file pone.0231789.s007.tiff]
